# A review of the pharmacology, clinical outcomes, and real-world effectiveness, safety, and non-contraceptive effects of NOMAC/E2

**DOI:** 10.1016/j.eurox.2024.100283

**Published:** 2024-01-22

**Authors:** Franca Fruzzetti, Rogerio Bonassi Machado, Iñaki Lete, Amisha Patel, Mitra Boolell

**Affiliations:** a Clinica San Rossore, Viale delle Cascine, 152/f, 56122 Pisa, Italy; b Faculty of Medicine of Jundiai, Jundiai, R. Francisco Telles, 250, 13202-550 Jundiaí, São Paulo, Brazil; c Jose Atxotegi Kalea, s/n, 01009 Gasteiz, Araba, Spain; d University Hospital Araba, s/n, 01009 Gasteiz, Araba, Spain; e Theramex HQ UK Ltd, 50 Broadway, 5th Floor, London SW1H 0BL, UK

**Keywords:** NOMAC/E2, Combined oral contraceptive, Real-world effectiveness, Venous thromboembolism, Menstrual bleeding

## Abstract

Selecting an appropriate oral contraceptive can be challenging for healthcare professionals due to the abundance of marketed contraceptive options with different clinical and real-world effectiveness and safety profiles. Nomegestrol acetate + 17β-estradiol (NOMAC/E2) is a combined oral contraceptive (COC) that inhibits ovulation by suppressing ovarian function by a 17-hydroxy-progesterone derivative and an estrogen identical to that endogenously produced by the ovaries. This narrative review examines clinical and real-world studies of NOMAC/E2 based on a background literature search using PubMed and Google Scholar. The review outlines the pharmacology of NOMAC/E2, including its progestational activity, pharmacokinetics, and effects on carbohydrate metabolism, lipid metabolism, and coagulation parameters, and summarizes key clinical efficacy and safety data that led to the approval of NOMAC/E2 in Europe, Brazil, and Australia. To help elucidate how NOMAC/E2 clinical trial data translate into a real-world setting, this review describes the effectiveness and safety of NOMAC/E2 in prospective studies that include over 90,000 users (half of whom received NOMAC/E2), outlining its effects on risk of thrombosis, menstrual bleeding patterns, weight, mood, acne, bone health, and patient quality of life. Non-contraceptive benefits of NOMAC/E2 for women with endometriosis, dysmenorrhea, or pre-menstrual dysphoric disorder are also discussed. These data demonstrate that NOMAC/E2 has a long half-life and rapid absorption, is effective at preventing unwanted pregnancies, and exhibits a favorable safety profile in both clinical trials and real-world settings. Importantly, NOMAC/E2 is not associated with increased risk of venous thromboembolism, a major safety concern of healthcare professionals for women receiving hormonal contraceptives. This review highlights NOMAC/E2 as a differentiated option among COCs and could help inform oral contraceptive choice to ultimately improve patient management and outcomes in real-world settings.

## Highlights

• Selecting an appropriate oral contraceptive can be challenging.
• NOMAC/E2 is effective at preventing pregnancy and shows a favorable safety profile.
• This review highlights NOMAC/E2 as a differentiated option among COCs.

## Introduction

The approval of the first oral contraceptive in 1960 represented a major achievement in the field of gynecology and sexual health. Over subsequent years, advancements in product dose and composition have led to the development of an array of effective oral contraceptive options [1]. The availability of reliable contraception has greatly reduced the risk of unwanted pregnancy and has helped prevent casualties from illegal abortion and childbirth. Contraception has helped women worldwide improve their quality of life (QoL) by providing the opportunity to choose the timing and number of their pregnancies [1].

Currently available oral contraceptives include the combined estrogen + progestin and progestin-only pills, both of which vary in formulation, dose, and regimen [2]. However, there is a need to gain a better understanding of the efficacy and safety profile of oral contraceptives when they are used in real life practice. This narrative review will focus on nomegestrol acetate + 17β-estradiol (NOMAC + E2; hereafter referred to as NOMAC/E2) as a differentiated option among combined oral contraceptives (COCs).

NOMAC/E2 was approved as an oral contraceptive by the European Medicines Agency in 2011 [3]. It is a COC comprising of 2.5 mg NOMAC, a 17-hydroxy-progesterone derivative, and 1.5 mg E2, an estrogen identical to that endogenously produced by the ovaries [3]. NOMAC/E2 follows a monophasic 24/4 regimen, comprising 24 days of an active pill and 4 days of a placebo pill [3]. The 4-day hormone-free period is enabled by the durable progestin activity of NOMAC, which has a half-life of approximately 46–50 h [4], [5].

COCs containing the synthetic progestins levonorgestrel or norethisterone in combination with ethinylestradiol (EE) at levels of ≤ 35 µg are generally considered first-line contraceptive options as they are associated with lower risks of venous thromboembolism (VTE) compared with other formulations [6]. However, given the abundance of marketed COCs, selecting an appropriate product can be challenging for healthcare professionals. This article aims to help optimize the management and outcomes of COC users by discussing key pharmacological, clinical, and real-world evidence on the benefits and drawbacks of NOMAC/E2.

## Methods

This review examines European pivotal and real-world studies of NOMAC/E2, including analyses from randomized controlled trials (RCTs), observational studies, and review articles. Case reports, commentaries, editorials, and congress abstracts were excluded. A background literature search focusing on NOMAC/E2 was conducted using PubMed and Google Scholar (last search date: September 2023) and the findings pooled from over 50 articles are discussed in this review.

## Factors to consider when prescribing a COC

Contraceptive counselling should involve the provision of evidence-based information on the safety, efficacy, advantages, and disadvantages of all methods of contraception. This enables women to make contraceptive choices based on their personal preferences and medical suitability. It is recommended that healthcare professionals prescribe a COC with demonstrated efficacy, based on available clinical and real-world evidence [6]. It should be noted that efficacy of COCs can be reduced by poor adherence [7]. COCs should also have the lowest possible effective dose of estrogen and progestogen, are well tolerated, have a favorable safety profile, offer additional non-contraceptive benefits if desired, and be affordable [6].

COCs are generally associated with a small risk of VTE [8]. Selection of a COC should include assessment of individual patient risk factors for VTE, including age, smoking, body mass index (BMI), immobilization, and a personal or family history of thromboembolism or thrombogenic mutations [6]. Women with a significant risk factor for VTE are not eligible for COCs [6].

The risk of other adverse events, such as bleeding between cycles, mood change, and weight gain [6], should be considered on an individual case basis when selecting a COC.

## Pharmacology

### Progestational activity and inhibition of ovulation

NOMAC is a synthetic 19-nor-progesterone derivative [9]. It is a progesterone receptor (PR) agonist that almost exclusively binds to progesterone receptors in hormone-sensitive cells derived from rats and humans [9]. NOMAC has a higher progestational potency than the synthetic progestin medroxyprogesterone acetate (MPA) due to the addition of a double bond in the C6–7 position and the deletion of the CH3 radical in position C19 of the hydroxyprogesterone skeleton [5].

Oral administration of NOMAC strongly inhibits ovulation in rats and monkeys (50% inhibitory dose [ID50] of 1.25 mg/kg and 0.14 mg/kg, respectively) [10]. NOMAC demonstrates strong affinity for the PR and exhibits high antigonadotrophic activity and moderate anti-androgenic properties [11]. NOMAC does not exhibit activity on estrogen, androgen, glucocorticoid, or mineralocorticoid receptors [11] and does not interfere with the beneficial effects of E2 [12].

In women, the combination of NOMAC and E2 inhibits ovulation by suppressing ovarian function [13]. E2 reduces sex hormone-binding globulin, transcortin, and angiotensinogen 600-, 500-, and 350-fold, respectively, versus the synthetic estrogen EE generally used in COCs [14].

### Pharmacokinetics, bioavailability, and metabolism

NOMAC/E2 has a pharmacokinetic profile consistent with once-daily dosing [4]. In humans, the absolute bioavailability of NOMAC and E2 is 63.4% and 1%, respectively [15]. NOMAC is metabolized by the hepatic cytochrome system enzymes CYP3A3, CYP3A4, and CYP2A6 and is excreted via the urine and feces [9]. In healthy women aged 18–50 years treated with daily NOMAC/E2 (n = 23), peak plasma concentration (12.3 ± 3.5 ng/mL) was reached in 1.5 h and steady state was achieved after 5 days of dosing. In this study, the mean ± standard deviation (SD) elimination half-life of NOMAC was 45.9 ± 15.3 h [4].

### Carbohydrate and lipid metabolism

NOMAC/E2 exhibits less influence on carbohydrate metabolism than COCs containing levonorgestrel (LNG)/EE [15] and has a neutral effect on lipid metabolism [16]. A Finnish randomized open-label study compared the effects of NOMAC/E2 (n = 60) and LNG/EE (150 μg/30 μg; n = 61) on carbohydrate metabolism in women aged 18–50 years. After six cycles, oral glucose tolerance tests demonstrated lower impact on carbohydrate metabolism with NOMAC/E2 versus LNG/EE (*P* ≤ 0.002), and only negligible changes from baseline in glucose (6.1% vs 13.9%, respectively [*P* = 0.02]) and insulin (–0.9 vs 26.4, respectively [*P* < 0.001]) parameters [17]. No clinically relevant changes were observed in total cholesterol, high-density lipoprotein C (HDL-C), low-density lipoprotein C (LDL-C), or total triglycerides in NOMAC/E2 users, while decreases in HDL-C and increases in LDL-C and total triglycerides were observed in LNG/EE users [17]. Similar results were observed in an observational cohort study of women with primary dysmenorrhea who received NOMAC/E2 (n = 20) or chlormadinone acetate/EE (CMA/EE; n = 40). NOMAC/E2 did not induce changes to lipoprotein or apoprotein levels versus baseline, whereas CMA/EE led to increased total cholesterol (*P* = 0.0114), HDL-C (*P* = 0.0008), triglycerides (*P* = 0.002), apoprotein-A1 (*P* = 0.0006), and apoprotein-B (*P* = 0.008), and a lower LDL/HDL ratio (*P* = 0.024) [18].

### Effects on coagulation parameters

Evidence suggests that NOMAC/E2 has a lower adverse effect on hemostasis than LNG/EE [15]. In 90 women aged 18–38 years, mean changes from baseline in prothrombin fragments 1 + 2 did not increase with NOMAC/E2 versus LNG/EE after three cycles. Significant mean differences between NOMAC/E2 and LNG/EE were observed for anti-thrombin, activated protein C resistance normalized ratio, D-dimer, plasminogen, and plasminogen activator inhibitor-1 [19].

In another study, NOMAC/E2 (n = 60) and LNG/EE (n = 61) induced minimal changes over six cycles in pro-coagulatory factors II, VIIa, and VIII, whereas factor VIIc was increased with NOMAC/ E2 and decreased with LNG/EE. Prothrombin fragment 1 + 2 concentrations were slightly increased with LNG/EE but not NOMAC/E2 [17]. It should also be noted that, after 6–13 cycles of NOMAC/E2, no mean changes from baseline were reported for either systolic or diastolic blood pressure [15].

## Clinical efficacy and safety

Various studies, which reflect optimal treatment conditions, contributed to the approval of NOMAC/E2. A double-blind, randomized, phase IIa dose-finding study of healthy pre-menopausal women aged 18–35 years assessed the ability of different doses of NOMAC/E2 (0.625 mg/1.5 mg [n = 9], 1.25 mg/1.5 mg [n = 10], 2.5 mg/1.5 mg [n = 10] or 2.5 mg NOMAC alone [n = 9]) to inhibit ovulation. After treatment for 21 days, 2.5 mg NOMAC inhibited ovulation and follicular maturation, with the antigonadotrophic effect reinformed by 1.5 mg E2. No serious adverse events (AEs) or significant differences in the incidence of AEs were observed between treatment groups [20]. Another study compared 24-day and 21-day regimens and demonstrated that the 24-day regimen promoted greater inhibition of follicular growth than the 21-day regimen, suggesting that NOMAC/E2 administered with the shorter pill-free interval assures a greater contraceptive efficacy [21]. A randomized, six-cycle study investigating the effects of NOMAC/E2 (n = 32) versus drospirenone/EE (DRSP/EE) (n = 16) on ovarian activity confirmed that NOMAC/E2 can consistently inhibit ovulation and suppress ovarian activity at a similar level to DRSP/EE [13].

Prior to approval, the efficacy of NOMAC/E2 was evaluated in two key clinical phase III trials: SAMBA (N = 2126) was conducted in Europe, Asia, and Australia, and RUMBA (N = 2200) was conducted in North, Central, and South America [22], [23]. The latter will not be discussed in this review.

In SAMBA, healthy, sexually active women aged 18–50 years received NOMAC/E2 (n = 1591) or DRSP/EE (n = 535; 21/7-day regimen) for 13 cycles. The estimated Pearl index (PI) among women aged 18–35 years was 0.38 (95% confidence interval [CI]: 0.10–0.97) with NOMAC/E2 (n = 1613) versus 0.81 (95% CI: 0.17–2.35) with DRSP/EE (n = 539). Notably, NOMAC/E2 users were allowed to miss one active tablet at any time and two active tablets mid-cycle (Days 8–17) without back-up requirement, while for DRSP/EE users, one missed active tablet during the first or third week, or two or more active tablets at any time, required the use of back-up contraception. For users ≤ 35 years of age, contraceptive efficacy of NOMAC/E2 appeared independent of age, race, smoking status, weight, and BMI. NOMAC/E2 demonstrated acceptable cycle control and was well tolerated, with a safety profile similar to that of DRSP/EE. Treatment-related adverse events (TRAEs) were experienced by 51.2% of women receiving NOMAC/E2 and 37.0% of women receiving DRSP/EE. Scheduled withdrawal bleedings were shorter and lighter with NOMAC/E2 than DRSP/EE and were sometimes absent altogether [23].

Recent studies may indicate a small risk of breast cancer in women taking COCs [24], [25], as stated in the prescribing information of NOMAC/E2 [3]. However, there is no clinical evidence to date suggesting such a risk is associated with NOMAC/E2 [26], [27], [28]. An in vitro study showed that E2 has a proliferative effect on ZR75–1 and HCC1500 breast cancer cell lines, however, this effect was inhibited by the accompanying progestogen [29]. In another in vitro study, the E2/NOMAC combination significantly reduced breast cell proliferation compared with E2 alone (p < 0.01) [30], perhaps because NOMAC has antiproliferative effects on breast cancer cells [31]. The antiproliferative effects of NOMAC/E2 could be an important point to consider when selecting a COC in women at risk of breast cancer.

## Nomac/E2 in a real-world setting

### Effectiveness

The multinational, controlled, prospective, active surveillance study PRO-E2 assessed the occurrence of unintended pregnancies in 91,313 new users of NOMAC/E2 (starters and re-starters) in Europe, Australia, and Latin America. Participants were randomized to receive NOMAC/E2 (n = 44,559) or LNG-containing COCs (COC_LNG_) (n = 46,754). The authors reported a significantly lower risk of unintended pregnancy with NOMAC-E2 compared with COC_LNG_ (*P* < 0.0001; PI of 0.15 [95% CI: 0.11–0.19] and 0.41 [95% CI: 0.35–0.47], respectively). Of note, women in the NOMAC/E2 cohort had a higher mean age (31.0 ± 8.6 years) than those in the COC_LNG_ cohort (29.3 ± 8.53 years) [32].

The impact of age on NOMAC/E2 contraceptive failure in PRO-E2 was subsequently investigated by stratifying women by age (<25 years and >40 years) [33], [34]. In women aged < 25 years, the risk of unintended pregnancy was significantly lower with NOMAC/E2 (n = 12,829) than with COC_LNG_ (n = 17,095), with PIs of 0.24 (95% CI: 0.16–0.35) and 0.51 (95% CI: 0.41–0.62), respectively [34]. In women aged > 40 years, unintended pregnancy did not differ substantially between the NOMAC/E2 (n = 7762) and COC_LNG_ (n = 6059) users (PI 0.08 [95% CI: 0.03–0.18] and PI 0.08 [95% CI: 0.03–0.18], respectively). The authors concluded that NOMAC/E2 assures a higher protection from unwanted pregnancy in young women but may represent a valid alternative to COC_LNG_ in all women, from adolescence to perimenopause [33], [34].

The durable effectiveness of NOMAC/E2 is partly attributed to its long half-life (approximately 46–50 h) [4], [5] and 24/4 regimen [21]. An estimated 15–51% of oral contraceptive users forget one to three pills per cycle [35], but the SAMBA study demonstrated that the contraceptive efficacy of NOMAC/E2 may be maintained even when one active tablet is missed at any time and two active tablets are missed mid-cycle (Days 8–17) [23].

### Bleeding patterns

The 24/4 regimen of NOMAC/E2 may significantly impact endometrial bleeding patterns, reduce menstrual flow, and induce favorable bleeding control [21]. Clinical studies suggest that NOMAC/E2 users have shorter, lighter scheduled bleeding than women receiving other COCs, such as DRSP/EE, or an absence of scheduled bleeding [15].

A pooled analysis of two open-label, randomized studies compared NOMAC/E2 (n = 2835) and DRSP/EE (3 mg/30 μg; n = 938) on bleeding patterns in healthy women aged 18–50 years. Women receiving NOMAC/E2 had a higher prevalence of absent scheduled bleeding (no bleeding episode that began during or continued into the expected bleeding period) versus DRSP/EE (17.6–31.6% versus 3.4–5.8% per cycle, respectively; *P* < 0.0001). Unscheduled bleeding (any bleeding episode occurring during the expected non-bleeding period) frequency decreased from Cycle 2 to 12 in both groups (from 23.2% to 15.4% and 17.4% to 10.9% in NOMAC/E2 and DRSP/EE users, respectively). Both regimens led to increases in amenorrhea (no bleeding/spotting during a 28-day cycle) over time, but amenorrhea was higher with NOMAC/E2 than DRSP/EE (*P* < 0.0001). In NOMAC/E2 users, both absent scheduled bleeding and amenorrhea were more common in older women, overweight women, switchers (prior hormonal contraceptive use), and smokers, whereas unscheduled bleeding was more common in starters (no prior hormonal contraceptive use) but was not associated with age, BMI, or smoking status [36].

### Weight, mood, and acne

In PRO-E2, changes in mood, acne, and weight were investigated in NOMAC/E2 and COC_LNG_ users aged < 25 years. Patients were asked acne-related questions at baseline and follow-up, and responses were scored and summarized into acne and mood scores scaled from 0 to 100 (higher scores indicated a better skin condition or mood) [34].

The majority of continuous NOMAC/E2 and COC_LNG_ users demonstrated minor changes (increase or decrease) in body weight (between 0 and <5%), mood score (between 0 and <25%), and acne score (between 0 and ≤ 25%) from baseline to 12-month follow-up. After 24 months, no differential effect on acne, mood, or weight was observed between NOMAC/E2 and COC_LNG_ users [34].

Changes in mood and weight were also monitored in users aged > 40 years, with no differential effect reported between cohorts from baseline through 24 months [33].

### Bone health

Real-world data on the effects of NOMAC/E2 on bone health are limited. A prospective, randomized, open-label study compared the effects of NOMAC/E2 (n = 56) and LNG/EE (150 μg/30 μg; n = 54) on bone mineral density (BMD) in women aged 20–35 years. No difference in BMD was reported between cohorts after 2 years, and the effect of NOMAC/E2 on BMD was not considered clinically relevant [37]. Further investigation is required to assess whether NOMAC/E2 treatment has an impact on bone health.

### Thrombosis risk

NOMAC/E2 may be a promising contraceptive option to reduce residual VTE risk associated with COCs. The risk of VTE (specifically deep venous thrombosis of the lower extremities and pulmonary embolism) and arterial thromboembolism (ATE) were compared in women receiving NOMAC-E2 (n = 44,559) and COC_LNG_ (n = 46,754) in PRO-E2. The incidence of VTE per 10,000 woman-years (WY) was similar with NOMAC/E2 and with COC_LNG_ (VTE: 2.5 [95% CI: 1.3–4.3] and 3.7 [95% CI: 2.3–5.7], respectively; ATE events WY: 0.8 [95% CI: 0.2–2.1] and 1.3 [95% CI: 0.5–27], respectively) [8]. Of particular interest, similar VTE incidence rates were reported for NOMAC/E2 and COC_LNG_ in women aged > 40 years (5.9 [95% CI: 1.9–13.7] and 5.9 [95% CI: 1.6–15.1] per 10,000 WY, respectively) [33].

### Patient experience

NOMAC/E2 is generally associated with high rates of patient satisfaction. The BOLERO study assessed the probability of treatment continuation and satisfaction in pre-menopausal women aged 18–50 years. Of 292 women evaluated, 73.7% (95% CI: 68.0–78.5%) continued treatment through 12 months and user satisfaction with NOMAC/E2 increased from 56.9% at initial evaluation to 89.2% at 12-month follow-up [16]. However, discontinuation rates from NOMAC/E2 treatment in some clinical trials suggest that some patients experienced low levels of satisfaction. In the PRO-E2 study, 15.0% of NOMAC/E2 users dropped out, compared with 16.3% of COC_LNG_ users [32]. A pooled analysis of two studies comparing NOMAC/E2 and DRSP/EE reported discontinuation rates of 34.6% and 30.8%, respectively [36].

## Non-contraceptive effects

### Management of endometriosis and dysmenorrhea

Women with endometriosis are often prescribed COCs [38]. In the Italian study of NOMAC/E2 (n = 99) versus dienogest (DNG) (n = 98) in women with endometriosis-associated chronic pelvic pain (CPP), improvement in CPP (as measured by visual analogic scale [VAS] score) was observed in both treatment groups from baseline through 12 months (*P* < 0.001). DNG led to a greater improvement in VAS score than NOMAC/E2 (*P* = 0.01; Cohen’s d=0.7) from baseline at 6 months, but not at 3 months (*P* = 0.06; Cohen’s d=0.8) or 12 months (*P* = 0.06; Cohen’s d=0.7). QoL improved from baseline to 12 months for both NOMAC/E2 and DNG (*P* < 0.001), as determined using the 36-Item Short Form Survey (SF-36) questionnaire. Better somatic scores were reported with DNG versus NOMAC/E2 at 6 (*P* = 0.01) and 12 months (*P* < 0.006). No women who received NOMAC/E2 or DNG reported to be dissatisfied or very dissatisfied during treatment [39].

In a retrospective cohort study of 39 women with pelvic endometriosis, NOMAC/E2 significantly reduced the global VAS score for pain symptoms associated with pelvic endometriosis from baseline through 6 months (8.1 versus 2.5 points [*P* = 0.0038]). NOMAC/E2 also decreased the size of ovarian endometriomas in 91.7% of participants, suggesting that NOMAC/E2 could be used to manage symptoms of pelvic endometriosis [40].

COCs are the preferred contraceptive therapy for dysmenorrhea, which affects up to 90% of women of reproductive age [18]. In a prospective, observational, cohort study of women with primary dysmenorrhea receiving NOMAC/E2 (n = 15) or CMA/EE (n = 19), only NOMAC/E2 was associated with significantly increased SF-36 scores (*P* = 0.001) in both the physical (*P* = 0.001) and mental (*P* = 0.004) domains.

A prospective, observational, single-center study investigated the effect of NOMAC/E2 (n = 15) and CMA/EE (21/7 regimen; n = 19) in women with primary dysmenorrhea requiring an oral contraceptive. Both NOMAC/E2 and CMA/EE significantly reduced the VAS score of primary dysmenorrhea (74.7% and 78.4%, respectively; *P* < 0.0001). The duration of menstrual bleeding was significantly reduced with NOMAC/E2 versus baseline (to 2.64 ± 1.59 versus 4.86 ± 1.20 days [*P* = 0.0005]) and versus CMA/EE users (*P* = 0.007). Regarding QoL, only NOMAC/E2 was associated with significantly increased SF-36 scores (*P* = 0.001) in both the physical (*P* = 0.001) and mental (*P* = 0.004) domains [18].

### Pre-menstrual symptoms and dysphoric disorder

Pre-menstrual symptom (PMS) is experienced by up to 80% of women of reproductive age. COCs are often used to treat pre-menstrual dysphoric disorder, a severe form of PMS that affects 2–5% of women and is associated with mood symptoms that cause clinically significant distress [41].

The effect of pre-menstrual and menstrual symptoms in healthy women receiving NOMAC/E2 (n = 2631) and DRSP/EE (n = 891) was assessed in a comparative analysis of two randomized, open-label, multicenter studies. Using the Mood Menstrual Distress Questionnaire Form C, significant improvement in “pain”, “water retention”, “negative affect”, “impaired concentration”, and “behavior change” domain scores were observed with NOMAC/E2 versus DRSP/EE (*P* = 0.001 for all comparisons) over the course of 13 consecutive 28-day cycles, suggesting that NOMAC/E2 is successful at reducing pre-menstrual and menstrual symptoms [42]. In addition, the phase IV ZOCAL study demonstrated higher QoL scores in women receiving COCs containing natural estrogen compared with those receiving COCs based on ethinylestradiol. Switching from COCs containing ethinylestradiol to natural estrogen was also associated with increased global and psychological QoL scores from baseline to 6 months, as determined using the Spanish Society of Contraception (SEC)-QOL scale [43].

In a randomized, placebo-controlled study of healthy women receiving NOMAC/E2 (n = 84) or placebo (n = 94) for three cycles, NOMAC/E2 was associated with small but significant increases in mean anxiety (0.22, 95% CI: 0.07–0.37; *P* = 0.003), irritability (0.23, 95% CI: 0.07–0.38; *P* = 0.012), and mood swing (0.15, 95% CI: 0.00–0.31; *P* = 0.047) scores during the inter-menstrual phase, as determined using the Daily Record of Severity of Problems questionnaire, but improvement in pre-menstrual depression (−0.33, 95% CI: −0.62 to −0.05; *P* = 0.049). The proportion of women who reported clinically relevant mood deterioration did not differ between NOMAC/E2 and placebo (24.1% and 17.0%, respectively; *P* = 0.262). NOMAC/E2 may cause small but statistically significant mood swings during the inter-menstrual phase in some women [44]. However, positive mood effects with NOMAC/E2 were noted in the pre-menstrual phase; the proportion of women with clinically relevant mood worsening did not differ between the treatment groups. These findings were driven by a subgroup of women who already suffer from COC-related side effects.

## Conclusions

The data discussed in this review support the use of NOMAC/E2 in sexually active women wishing to avoid pregnancy due to its long half-life, rapid absorption, favorable safety profile, and effectiveness at preventing unwanted pregnancies. Notably, NOMAC/E2 may be particularly useful in adolescents due to its maintained contraceptive efficacy when doses are missed [35]. In addition, real-world findings suggest incidence levels of VTEs, a major safety concern for healthcare professionals, with NOMAC/E2 similar to COCs containing levonorgestrel, which are believed to provide a low risk of VTE among COCs. Further research in real-world settings is required to strengthen the position of NOMAC/E2 as a first-line oral contraceptive option.

## Funding

The development of this manuscript was funded by Theramex (London, UK). Theramex contributed to the project initiation, literature research, critical revision of the manuscript, and decision to submit for publication.

## CRediT authorship contribution statement

**Boolell Mitra:** Writing – review & editing. **Fruzzetti Franca:** Writing – review & editing. **Machado Rogerio Bonassi:** Writing – review & editing. **Lete Inaki Luis Ignacio:** Writing – review & editing. **Patel Amisha:** Writing – review & editing.

## Declaration of Competing Interest

**FF**: None.

**RBM** has a financial relationship (lecturer or member of advisory boards) with Abbott, Bayer, Besins Healthcare, Gedeon Richter, Organon, Libbs, Novo Nordisk and Theramex.

**IL** worked in an advisory role with Kern Pharma Portugal.

**AP** is an employee of Theramex.

**MB** is an employee of Theramex.
